# Allergic contact dermatitis caused by 1,6‐hexanediol diacrylate in a hospital wristband

**DOI:** 10.1111/cod.13378

**Published:** 2019-08-28

**Authors:** Cynthia C. A. van Amerongen, Jakob Dahlin, Marléne Isaksson, Marie L. A. Schuttelaar

**Affiliations:** ^1^ Department of Dermatology University of Groningen, University Medical Center Groningen Groningen The Netherlands; ^2^ Department of Occupational and Environmental Dermatology Lund University, Skåne University Hospital Malmö Sweden

**Keywords:** 1,6‐hexanediol diacrylate (1,6‐HDDA), acrylate, allergic contact dermatitis, CAS no. 13048‐33‐4, case report, wristband

## Abstract

**Background:**

1,6‐Hexanediol diacrylate (1,6‐HDDA) is a multifunctional acrylate and a potent sensitizer.

**Objectives:**

To report a case of allergic contact dermatitis caused by 1,6‐HDDA in a hospital wristband.

**Methods:**

A male patient presented with eczema on his wrist where he had worn a hospital wristband. Patch testing was performed with our extended European baseline series, additional series, and pieces of the hospital wristband. Thin‐layer chromatography (TLC) was performed with extracts from the wristband and gas chromatography‐mass spectrometry was used for chemical analysis.

**Results:**

Positive reactions were found to pieces of the wristband, including adhesive rim (+++), inside (+++), and outside (++); to multiple allergens in the (meth)acrylates series; and to extracts of the wristband in acetone and ethanol. Chemical analysis of the ethanol extract showed presence of lauryl acrylate and 1,6‐HDDA. Patch testing with TLC strips and subsequent chemical analysis showed that the substance causing the strongest reaction was 1,6‐HDDA, to which the patient had a confirmed positive patch test reaction.

**Conclusion:**

1,6‐HDDA was identified as the culprit allergen responsible for allergic contact dermatitis caused by the hospital wristband.

## 1 INTRODUCTION

Patient identification (ID) wristbands are widely used in hospitals to provide patient information, thereby reducing the risk of patient misidentification. We report a patient with allergic contact dermatitis caused by 1,6‐hexanediol diacrylate (1,6‐HDDA) in a hospital patient ID wristband.

## 2 CASE REPORT

**Figure 1:**
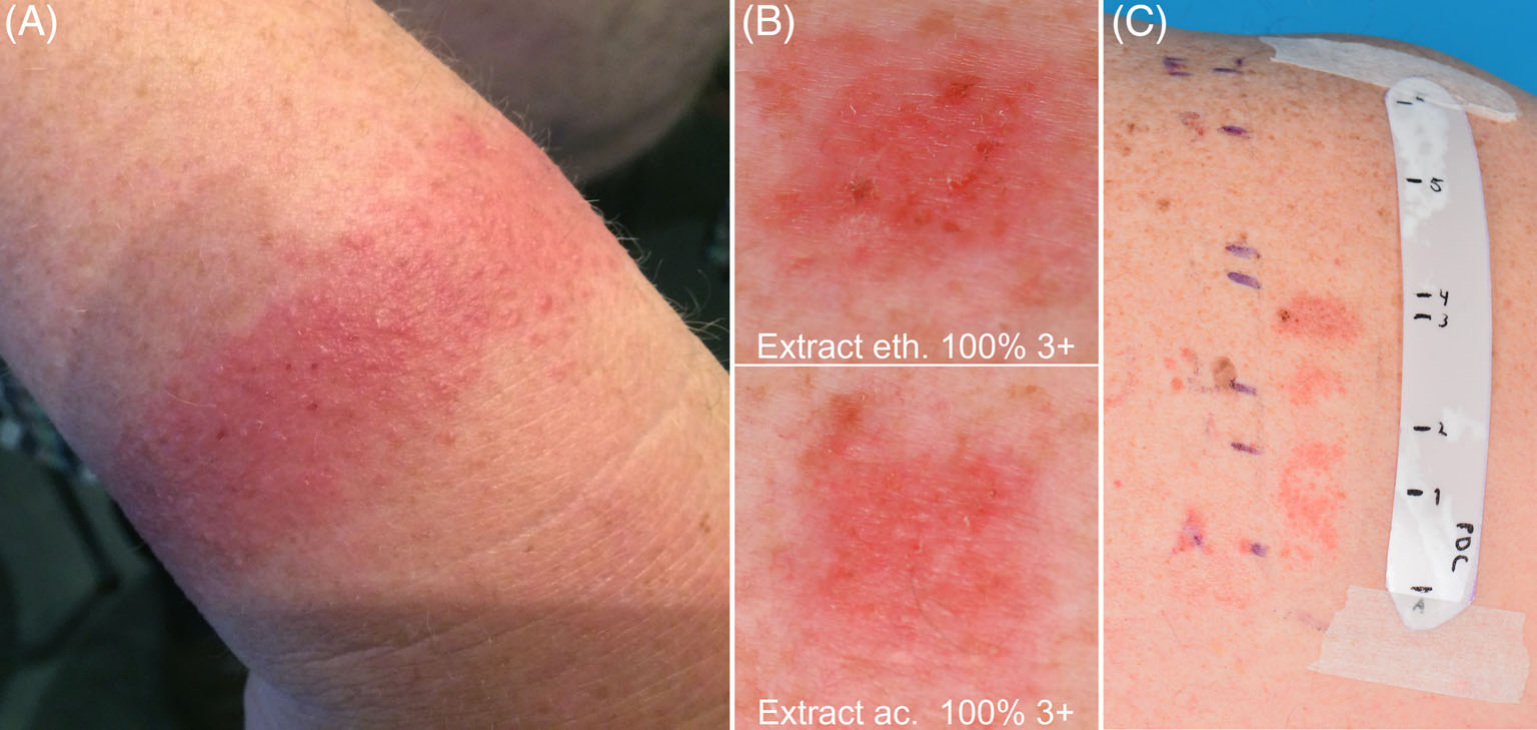
**(A**), Pruritic erythema and vesicles on the wrist where a DuraSoft hospital wristband was worn; (**B**), Positive patch test results to extracts at day (D) 3; (**C**), TLC testing: Positive reactions on areas to spot 3 (+++), spot 2 (++), and spot 1 (++) at D7

A 66‐year‐old non‐atopic man was referred to the Department of Dermatology for evaluation of pruritic erythema and vesicles on the wrist at the site where he wore a hospital ID wristband (DuraSoft Laser Patient ID system; Precision Dynamics Corporation [PDC] Healthcare, Valencia, California) during hospitalization due to cardiac failure (Figure 1A). The patient reported that the skin complaints on the wrist at the site of the wristband started a few hours after the wristband was put on. During a previous hospitalization, at which the same type of hospital wristband had been used, no skin complaints on the wrist were observed. He reported a history of similar skin complaints at the sites of application of electrocardiogram (ECG) electrodes. The patient was retired, and his previous job had been delivering parcels for 10 years. Before that he had been a butcher. He recalled possible relevant leisure exposure to two‐component adhesive in the past, but it was unclear whether the adhesive contained acrylates.

### 2.1 Patch testing

The patient was patch tested with our extended European baseline series (TRUE Test panels 1 and 2; supplemented with investigator‐loaded allergens) as well as cosmetics, fragrances, (meth)acrylates, and a plastics and glues series (allergens from SmartPractice Europe, Barsbüttel, Germany, and Chemotechnique Diagnostics, Vellinge, Sweden). Furthermore, pieces of the DuraSoft hospital wristband were tested “as is”. Because of the history of a skin reaction to ECG electrodes, various parts of these electrodes were also tested. Van der Bend Chambers were used (Van der Bend, Brielle, The Netherlands), which were fixed with Fixomull Stretch (BSN Medical, Hamburg, Germany). The patch tests were applied on the back for 48 hours under occlusion, and patch test readings were performed at day (D) 3 and D7, according to ESCD guidelines.^1^

Strong positive patch test reactions were found to pieces of the wristband: adhesive rim (+++), inside (+++), and outside (++). Multiple strong positive reactions to the (meth)acrylates series were seen. Furthermore, the patient showed positive reactions to the gel and adhesive layer of several ECG electrodes. Results are shown in Table 1.

**Table 1:** Patch test results from patch test series, wristband product, extracts, substances, and thin‐layer chromatography strips

Tested series, wristband product		Concentration, vehicle	D3	D7	
**Extended European baseline series**		μg/cm^2^			
*p‐tert*‐butylphenol formaldehyde resin (PTBP‐FR)		40	++	+	
**Plastic/glue series**		(%) pet.			
*p‐tert* butylphenol		1	++	+	
**(Meth)acrylate series**		(%) pet.			
Ethyl acrylate (EA)		0.1	+++	+++	
Butyl acrylate (BA)		0.1	+++	++	
2‐Hydroxyethyl acrylate (2‐HEA)		0.1	+++	+++	
2‐Hydroxypropyl acrylate (2‐HPA)		0.1	+++	+++	
2‐Hydroxyethyl methacrylate (2‐HEMA)		2	+	++	
2‐Hydroxypropyl methacrylate (2‐HPMA)		2	+++	++	
Triethylene glycol dimethacrylate (TEGDMA)		2	++	++	
1,4‐Butanediol dimethacrylate (BUDMA)		2	+++	++	
1,4‐Butanediol diacrylate (BUDA)		0.1	+++	+++	
1,6‐Hexanediol diacrylate (HDDA)		0.1	+++	+++	
Diethylene glycol diacrylate (DEGDA)		0.1	+++	+++	
Tripropylene glycol diacrylate (TPGDA)		0.1	+++	+++	
Trimethylolpropane triacrylate (TMPTA)		0.1	+	++	
Pentaerythritol triacrylate (PETA)		0.1	+	++	
Oligotriacrylate (OTA)		0.1	+++	++	
Epoxy acrylate		0.5	++	++	
Triethylene glycol diacrylate (TREGDA)		0.1	+++	+++	
**Wristband product**					
Wristband adhesive part		“as is”	+++	++	
Wristband inside		”as is”	+++	++	
Wristband outside		“as is”	++	++	
**Wristband extracts**			**D2**	**D3**	**D7**
Extract wristband ethanol		100% eth.	++	+++	++
Extract wristband ethanol		10% eth.	−	+	?+
Extract wristband acetone		100% ac.	++	+++	++
Extract wristband acetone		10% ac.	−	+	?+
**TLC**					
TLC below spot 3 a			+++	+++	+++
TLC spot 2			−	++	++
TLC spot 1			−	+	++
**Substances identified on TLC scrapings**		% pet.	**D3**	**D4**	**D7**
Isobornyl acrylate		0.1	?+	−	−
Isobornyl acrylate		0.01	?+	−	−
Lauryl acrylate		0.1	?+	?+	−
Lauryl acrylate		0.01	?+	−	−
**ECG electrodes**			**D3**	**D7**	
3 M Red Dot 2570	Gel	”as is”	++	+++	
3 M Red Dot 2249‐50	Adhesive layer	”as is”	++	++	
3 M Red Dot 2249‐50	Gel	”as is”	+++	++	
3 M Red Dot 2255	Adhesive layer	”as is”	++	+++	
3 M Red Dot 2255	Gel	”as is”	+++	+++	

TLC spots 4, 5, and E (solvent front) negative.

Abbreviations: ac., acetone; eth., ethanol; pet., petrolatum; TLC, thin‐layer chromatography.

a Used for chemical analysis.

### 2.2 Thin‐layer chromatography (TLC) and chemical analysis

Subsequently, extracts from the wristband were prepared in acetone and ethanol. Extraction was performed in an ultrasonic bath followed by concentration using a vacuum evaporator.^2^ When the patient was patch tested, positive reactions to the extracts of the wristband were observed in acetone undiluted (+++) and diluted to 10% (+) as well as in ethanol undiluted (+++) and diluted to 10% (+) (Figure 1B and Table 1). The tested ethanol extract was analysed with gas chromatography‐mass spectrometry (GC‐MS) and showed presence of lauryl acrylate and 1,6‐HDDA. The presence of these substances was confirmed by analysis of reference substances.

TLC was performed in order to separate the components of the extracts of the wristband material and to obtain TLC strips for patch testing.^3^ The spots, containing 65 μL of the undiluted ethanol extract, were applied to a sheet of thin‐layer material with silica gel bound to a plastic carrier (TLC plastic roll, Silica Gel 60F 254; Merck, Darmstadt, Germany). A mobile phase consisting of heptane and ethyl acetate (50:50, vol/vol) was used for elution.^3^ After elution, spots visible in UV‐radiation (254 and 366 nm) were marked with a pencil.

The patient was tested with the TLC strips (extract ethanol) and showed positive reactions below spot 3 (+++), spot 2 (++), and spot 1 (++) (Figure 1C and Table 1). Scrapings from reference TLC‐plates did not demonstrate the presence of lauryl acrylate and 1,6‐HDDA. Benzophenone could be identified in scrapings from spot 3. Eluting benzophenone together with higher concentrations of lauryl acrylate and 1,6‐HDDA in the same type of TLC system as used before, followed by chemical analysis with GC‐MS, revealed the presence of lauryl acrylate just above benzophenone, and 1,6‐HDDA just below benzophenone. Hence, with an indirect identification method the substance causing the strongest reaction for the DuraSoft wristband TLC (below spot 3) was identified as 1,6‐HDDA, for which the patient had a confirmed positive patch test reaction in earlier testing. The concentration of 1,6‐HDDA in the patch test extract was approximately 0.003%, corresponding to 19 μg/g in the wristband.

Separate analysis of concentrated extracts of the adhesive rim and the other part of the band was performed. No acrylates in any detectable amount could be detected in the adhesive rim. Analysis of the band showed that the present acrylates came from the glue used for fixation of the paper and plastic upper layer. The previously mentioned 1,6‐HDDA and lauryl acrylate, as well as traces of isobornyl acrylate, were found in these paper/plastic samples. Additional patch testing was performed with isobornyl acrylate 0.1% and 0.01% pet. and with lauryl acrylate 0.1% pet. and 0.01% pet., which all were negative.

## 3 DISCUSSION

We present a patient with allergic contact dermatitis caused by 1,6‐HDDA, which was probably a component of the glue used for fixation of the paper and plastic upper layer of his hospital wristband. 1,6‐HDDA (CAS no. 13048‐33‐4) is a multifunctional acrylate. These acrylates, which are components of printing inks and coatings, are also used in dentistry. Contact allergy is mostly seen after occupational exposure.^4^

The multiple positive reactions to (meth)acrylates in our patient can be explained by cross‐reactivity or co‐sensitization. The patient reacted to the gel and adhesive layer of the ECG electrodes. According to the ingredient list, 3 M Red Dot 2570 contained an acrylate glue and polyethylene glycol dimethacrylate, and 3 M Red Dot 2249‐50 and 3 M Red Dot 2255 contained an acrylate copolymer. Previous studies have shown that (meth)acrylates or acrylic acid were the culprit allergens in patients with allergic contact dermatitis caused by contact with ECG electrodes.^5^ Therefore co‐sensitization could be another explanation for multiple reactivity to various (meth)acrylates in our patient. The positive reactions to spot 1 and 2 of the TLC probably corresponded to other (meth)acrylates that could not be identified. The positive reaction to *p‐tert*‐butylphenol formaldehyde resin (PTBP‐FR) in our patient could also be explained by the ECG electrodes, which can contain this allergen in the adhesive layer and gel of the electrodes.^6^

In the current case, the acetone extract could not be tested with TLC because it was difficult to apply and did not produce good separations on the TLC; the wristband was made from vinyl, which contains a large number of plasticizers. Acetone dissolves many of these plasticizers, and their presence interferes with both application and separation of the sample. The limited presence of lauryl acrylate and 1,6‐HDDA on the reference TLC‐plates was probably because the concentration on the plates was too low.

A few cases of allergic contact dermatitis caused by wristbands have been reported in the literature.^7^, ^8^ Tanahashi et al reported three patients who wore the same type of amusement park wristband; positive photopatch test results to benzophenone 1% pet. were observed in the three cases and benzophenone was identified as the culprit allergen. Because 1,6‐HDDA was also detected by GC‐MS in the wristbands, patch testing was performed, but results were negative.^7^ Hills and Ive reported diisodecyl phthalate as the allergen responsible for allergic contact dermatitis in a patient who wore a polyvinyl chloride hospital ID wristband.^8^ It is interesting to note that the current case and the reported cases in the literature show that different contact allergens are responsible for allergic contact dermatitis caused by wristbands.

## CONFLICTS OF INTEREST

There was no funding and the authors report no conflicts of interest.
